# Association between perceived social support and post-traumatic growth among female thyroid cancer survivors: the chain mediating role of psychological resilience and coping strategies

**DOI:** 10.3389/fpsyg.2025.1614974

**Published:** 2025-10-06

**Authors:** Qing Lyu, Yinchu Hu, Cuimin Kou, Yan Li, Zhengjiang Li, Shaoyan Liu, Li Fu, Na Wang

**Affiliations:** ^1^Department of Head and Neck Surgical Oncology, National Cancer Center/National Clinical Research Center for Cancer/Cancer Hospital, Chinese Academy of Medical Sciences and Peking Union Medical College, Beijing, China; ^2^School of Nursing, Peking University, Beijing, China; ^3^School of Nursing, Liaoning University of Traditional Chinese Medicine, Shenyang, Liaoning, China; ^4^School of Nursing, Capital Medical University, Beijing, China

**Keywords:** post-traumatic growth, perceived social support, psychological resilience, coping strategies, female, thyroid cancer

## Abstract

**Introduction:**

The incidence of thyroid cancer has exhibited a steady upward trajectory, with women being more prevalent than men. Meanwhile, female thyroid cancer survivors are more prone to encountering negative psychological issues. Post-traumatic growth (PTG), a key element of positive psychology, has not been extensively studied in female thyroid cancer survivors, with unclear development pathways. This study aimed to investigate the correlates of PTG in female thyroid cancer survivors, including perceived social support, psychological resilience, and coping strategies.

**Methods:**

Six hundred and thirty-five female thyroid cancer survivors were included in this cross-sectional study. Data were collected using an online questionnaire comprising demographic information and scales measuring perceived social support, psychological resilience, coping strategies, and PTG. The relationships among variables were assessed using Pearson correlation analysis. The potential mediating roles were explored using AMOS 26.0. The indirect effects were assessed through bias-corrected bootstrapping procedure, utilizing 5,000 bootstrap samples.

**Results:**

Perceived social support was directly related to PTG (*p* < 0.001) in female thyroid cancer survivors. It exerted indirect effects on PTG through the mediating roles of specific coping strategies, including problem-focused coping (effect size = 0.197, *p* < 0.001), emotion-focused coping (effect size = 0.141, *p* < 0.001), and avoidant coping (effect size = 0.009, *p* < 0.05). In addition, chain mediation pathways involving both psychological resilience and coping strategies were identified. Specifically, a significant chain mediation effect was found through psychological resilience and emotion-focused coping (effect size = 0.008, *p* = 0.002), as well as through psychological resilience and avoidant coping (effect size = −0.003, *p* = 0.031). In contrast, psychological resilience alone did not serve a significant mediating role between perceived social support and PTG (*p* > 0.05).

**Conclusion:**

Psychological resilience and emotion-focused/avoidant coping strategies play a chain mediating role in the relationship between perceived social support and PTG in female thyroid cancer survivors. These findings enhance the understanding of the predictive effects of perceived social support on PTG and suggest ways to enhance PTG among female thyroid cancer survivors.

## 1 Introduction

Thyroid cancer has emerged as a significant global public health challenge, recognized as the most prevalent endocrine tumor worldwide. Epidemiological data reveal an alarming 4.77-fold increase in annual incidence from 123,000 cases in 2000 to 586,000 cases in 2020 (Parkin et al., 2001; Sung et al., 2021). Notably, women bear disproportionate burden, exhibiting 3-fold higher incidence rates compared to men (Pizzato et al., 2022). This gender-specific vulnerability creates unique psychological stressors. First and foremost, thyroid cancer predominantly affects women aged 20–39 years, a crucial period in their reproductive lives, which heightens fertility-related anxieties (Ginsburg et al., 2023). Furthermore, treatment-related issues such as scarring, voice changes, and metabolic dysregulation drive female body image dissatisfaction, further exacerbating their psychological distress (Shobab et al., 2022). What's more, despite a promising 5-year survival rate exceeding 90 % (Pizzato et al., 2022), survivors face persistent psychological distress from treatment sequelae like recurrent laryngeal nerve damage and permanent hypoparathyroidism (Noto et al., 2022). Considering the growing population of female thyroid cancer survivors and their elevated psychological burdens, healthcare professionals should prioritize the mental health of these individuals.

Post-traumatic growth (PTG), a pivotal concept in positive psychology, has attracted great attention to uncovering the beneficial impacts of traumatic events on individuals. PTG illuminates the constructive psychological metamorphoses individuals undergo following traumatic experiences encompassing changes in self-perception, interpersonal relationships, and life values (Tedeschi and Calhoun, 1996). Cancer survivors, for instance, have been found to not only experience negative emotions but also exhibit positive psychological responses such as heightened psychological resilience and personal strength (Ballinger et al., 2021). Furthermore, prior investigations indicated that PTG can serve as a predictor of quality of life among cancer survivors (Lim, 2019; Ofei et al., 2023). However, exploration of PTG among female thyroid cancer survivors remains limited (Misra et al., 2013), neglecting gender-specific PTG mechanisms. Therefore, it is imperative to investigate PTG in this cohort and understand potential mechanisms to provide insights into developing targeted interventions that transform survival advantages into holistic recovery.

The model of post-traumatic growth (PTG) highlights the trajectory individuals undergo following a traumatic event, encompassing challenges, rumination, contemplation, cognitive transformation, and personal growth (Tedeschi and Calhoun, 2004). This process is influenced by individual coping strategies and social support, although the exact relationship between them is not explicitly illustrated in the model (Tedeschi and Calhoun, 2004). Additionally, the life crisis and personal growth model suggests that an individual's characteristics play a role in the psychological consequences of life crises (Schaefer and Moos, 1998). Psychological resilience, an important trait reflecting one's ability to maintain a positive attitude and promptly recover from adversity, may serve as a potential antecedent to PTG. Although these two models pointed out the influencing factors of PTG, how these factors interacted with each other to promote the development of PTG among female thyroid cancer survivors was unclear.

Perceived social support is a crucial predictor of PTG in cancer survivors. The current evidence revealed a positive correlation between PTG and social support among adult cancer patients (*r* = 0.3) (Shand et al., 2015), with various types of social support, such as perceived availability of social support, playing key roles in predicting PTG in cancer patients (Schroevers et al., 2010; Scrignaro et al., 2011). Additionally, interventions like self-help groups have shown promise in enhancing PTG in breast cancer patients (Choi et al., 2022). Beyond direct effects, emerging evidence implicates psychological resilience and adaptive coping as mediators on PTG among cancer survivors (Chen et al., 2022; Ding et al., 2024; Dong et al., 2017). Notably, this mediation mechanism remains unexamined in female thyroid cancer populations, where gender-specific stressors (e.g., body image changes, fertility concerns) may uniquely influence PTG.

Coping strategies may be an important mediator in the relationship between perceived social support and PTG of cancer survivors. Coping strategies refer to specific actions or plans taken when facing problems, challenges, or crises, aimed at effectively addressing issues, mitigating impacts, or resolving risks. Previous studies have illustrated that social support was positively correlated with coping strategies in cancer patients, such as problem-solving, positive thinking, and hope (Cao et al., 2018; Chen et al., 2022). In terms of the link between coping strategies and PTG, findings from breast cancer survivors suggested that “Fatalism” coping strategies were significant predictors of PTG (Romeo et al., 2019). Additionally, research has shown that problem-focused coping strategies could significantly forecast long-term PTG in cancer patients (Scrignaro et al., 2011). More importantly, adaptive coping has been identified as a mediator between social support and PTG (Cao et al., 2018). Considering the above evidence and the established connections between coping strategies and social support and PTG, we proposed that coping strategies may play a mediating role in the association between perceived social support and PTG in female thyroid cancer survivors.

Psychological resilience is another indirect link between perceived social support and PTG of cancer survivors. Firstly, accumulated evidence indicated that psychological resilience was positively related to social support in a wide range of cancer survivors, such as bladder cancer (Dong et al., 2022), lung cancer (Yin et al., 2022), and breast cancer (Aizpurua-Perez et al., 2024; Ostadi-Sefidan et al., 2024). Furthermore, a recent meta-analysis reported that social support intervention could statistically significantly enhance resilience in cancer patients (Ding et al., 2024), indicating social support as a key predictor of psychological resilience. Regarding the relationship between psychological resilience and PTG, a study of colorectal cancer survivors indicated a significant positive association between resilience and PTG, and resilience was found to act as a mediator in the relationship between perceived social support and PTG (Dong et al., 2017). Additionally, numerous studies have highlighted the interplay between psychological resilience and coping strategies. For instance, quantitative research revealed a positive correlation between resilience, social support, and coping strategies among palliative patients facing advanced cancer (Lau et al., 2021). This positive correlation was also observed in liver cancer patients, with resilience accounting for 30.5 % of the variance in problem-focused strategies, 8.5 % in emotion-focused strategies, and 21.6 % in overall coping strategies (Chen et al., 2022). Overall, psychological resilience and coping strategies may represent a chain mediating pathway between perceived social support and PTG in female thyroid cancer survivors.

Therefore, this study aimed to explore the relationship between perceived social support and PTG and to elucidate the underlying mechanisms by which perceived social support affects PTG in female thyroid cancer survivors through psychological resilience and coping strategies. By shedding light on these mediators, we hope to provide valuable insights to facilitate PTG in this population. The hypotheses were shown as follows:

Hypotheses 1 (H1): Perceived social support can positively predict PTG in female thyroid cancer survivors.
Hypotheses 2 (H2): Psychological resilience mediates the relationship between perceived social support on PTG in female thyroid cancer survivors.
Hypotheses 3 (H3): Coping strategies mediate the relationship between perceived social support on PTG in female thyroid cancer survivors.
Hypotheses 4 (H4): Psychological resilience and coping strategies play a chain mediating role from perceived social support to PTG in female thyroid cancer survivors.

## 2 Methods

### 2.1 Study design and setting

This cross-sectional study was conducted on female patients who had received surgery for thyroid cancer at a university-affiliated oncology hospital in Beijing, China.

### 2.2 Participants

This study was conducted from 20th April 2023 to 20th October 2023, involving female thyroid cancer survivors. A convenience sampling method was adopted to recruit participants. Participants were included if they met the following criteria: (1) aged between 18 and 80 years old; (2) had undergone surgery for thyroid cancer; (3) possessed a device capable of accessing electronic questionnaires, such as a smartphone or tablet; and (4) voluntarily participated in the study and informed consent. Participants were excluded if they: (1) had a self-reported history of psychological disorders; (2) had severe cognitive impairment that could not guarantee the accuracy of the information; (3) suffered from other conditions that made them unfit to participate in the study, such as dyslexia, visual impairment, and disability; (4) withdrew during the survey or provided incomplete data. The sample size suggested for structural equation modeling (SEM) is that the ratio of the total sample size to parameters should be no less than 10:1 (Kline, 2015). Therefore, at least 490 cases were needed to test a model incorporating 49 free parameters in our study.

### 2.3 Measures

#### 2.3.1 Baseline characteristics and information of participants

The baseline characteristics and information of participants were assessed by a self-designed questionnaire that included a range of items: participants' age, ethnicity, religion, education level, employment status, monthly income, marital status, existing child/children, residence, current treatment status, time since first diagnosis, number of surgeries, pathologic subtypes, pathological staging, lateral neck dissection, and family history of thyroid cancer. The response options of the above questions are detailed in Table 1.

**Table 1:** Characteristics of the participants (*N* = 635).

**Characteristics**	***n (%)***	**PTG (mean ±SD)**	***t*/*F***	***p***
**Age**			5.305	**0.005**
18–44	395 (62.2)	29.94 ± 12.71		
45–59	199 (31.3)	26.65 ± 12.47		
≥60	41 (6.5)	26.10 ± 13.85		
**Ethnicity**			−1.378	0.174
Han	592 (93.2)	28.51 ± 12.97		
Others	43 (6.8)	30.74 ± 10.05		
**Religion**			−0.733	0.464
Yes	28 (4.4)	30.39 ± 12.04		
No	607 (95.6)	28.58 ± 12.84		
**Education level**			0.528	0.663
Junior high school and below	84 (13.2)	27.02 ± 11.77		
High school/junior college	134 (21.1)	28.93 ± 12.79		
Bachelor degree	356 (56.1)	28.92 ± 13.07		
Postgraduate and above	61 (9.6)	28.80 ± 12.72		
**Employment**			0.276	0.759
**status**				
Full-time employed	409 (64.4)	28.92 ± 12.98		
Part-time employed	44 (6.9)	28.61 ± 11.10		
Unemployed/retired	182 (28.7)	28.08 ± 12.82		
**Monthly**			0.169	0.917
**income (RMB)**				
< 1,000	77 (12.1)	29.51 ± 12.69		
1,000–5,000	226 (35.6)	28.77 ± 11.85		
5,000–10,000	217 (34.2)	28.45 ± 13.16		
≥10,000	115 (18.1)	28.29 ± 14.05		
**Marital status**			0.697	0.498
Legally married	533 (83.9)	28.82 ± 12.93		
Unmarried	80 (12.6)	27.21 ± 12.60		
Divorced	22 (3.5)	30.14 ± 10.14		
**Number of**			0.246	0.806
**children**				
0	127 (20.0)	28.41 ± 12.45		
≥1	508 (80.0)	28.72 ± 12.90		
**Residence**			0.192	0.848
Local	129 (20.3)	28.85 ± 13.43		
Non-local	506 (79.7)	28.61 ± 12.65		
**Current**			2.138	0.094
**treatment status**				
Hospitalized in surgery	234 (36.9)	29.32 ± 12.56		
Regular offline clinic reviews	317 (49.9)	28.78 ± 12.77		
Regular Internet clinic reviews	63 (9.9)	27.63 ± 13.54		
Without any treatment	20 (3.1)	22.05 ± 12.73		
**Time since first**			3.201	**0.023**
**diagnosis**				
< 6 months	322 (50.7)	30.07 ± 12.72		
6 months−2 years	195 (30.7)	27.61 ± 12.42		
2 years−5 years	81 (12.8)	27.40 ± 13.38		
≥5 years	37 (5.8)	24.73 ± 13.04		
**First surgery**			−0.206	0.837
Yes	575 (90.6)	28.63 ± 12.70		
No	60 (9.4)	28.98 ± 13.79		
**Pathologic**			0.254	0.776
**subtypes**				
Papillary carcinoma	626 (98.6)	28.70 ± 12.82		
Follicular carcinoma	4 (0.6)	27.25 ± 7.14		
Medullary carcinoma	5 (0.8)	24.80 ± 14.53		
**AJCC staging**			0.305	0.822
1	537 (84.6)	28.53 ± 12.65		
2	35 (5.5)	29.54 ± 13.89		
3	3 (0.5)	34.67 ± 10.07		
Missing	60 (9.4)	28.98 ± 13.79		
**Lateral neck**			−0.954	0.340
**dissection**				
Yes	271 (42.7)	29.22 ± 12.56		
No	364 (57.3)	28.24 ± 12.98		
**Family history of**			−1.536	0.125
**thyroid cancer**				
Yes	156 (24.6)	30.03 ± 12.68		
No	479 (75.4)	28.22 ± 12.82		

The bold values refer to the statistically significant items.

#### 2.3.2 Perceived social support

The abbreviated version of the Medical Outcomes Study Social Support Scale (MOSSS-5) was used to measure the level of perceived social support of female thyroid cancer survivors (Sherbourne and Stewart, 1991). The MOSSS-5 comprises five items, each rated on a 5-point Likert scale. The total score on the MOSSS-5 ranges from 5 to 25, with a total score between 5 and 16 indicating a low level of perceived social support. The MOSSS-5 has been validated and tested in Chinese populations, demonstrating acceptable internal consistency (Cronbach α coefficients of 0.91) and test-retest reliability (intraclass correlation coefficients of 0.89) (Wang et al., 2013).

#### 2.3.3 Psychological resilience

The Chinese version of the Brief Resilience Scale (BRS) was utilized to measure the psychological resilience of female thyroid cancer survivors, reflecting an individual's capacity to recover from stress. The BRS comprises six items rated on a scale from 1 (strongly disagree) to 5 (strongly agree). Mean scores for all six items are categorized as indicating low resilience (1–2.99), normal resilience (3-4.30), or high resilience (4.31–5) (Smith et al., 2008). The Chinese version of the BRS showed acceptable internal consistency, as evidenced by Cronbach's α value of 0.71 (Fung, 2020).

#### 2.3.4 Coping strategies

Coping strategies were measured using Brief Coping Orientation to Problem Experienced (COPE) inventory developed by (Carver 1997). The Brief-COPE comprises 28 items grouped into 14 factors, with each factor consisting of two items. The coping strategies in the Brief-COPE are categorized into problem-focused, emotion-focused, and avoidant coping strategies (Cooper et al., 2006). Responses are rated on a 4-point Likert scale, ranging from 1 (I have not been doing this at all) to 4 (I have been doing this a lot). The Chinese version of the Brief-COPE has been utilized in caregivers of stroke survivors and showed satisfactory internal consistency with a Cronbach's α value of 0.83 (Qiu and Li, 2008).

#### 2.3.5 Post-traumatic growth

The Short Form of Post-Traumatic Growth Inventory (PTGI-SF) developed by Cann et al. was used to measure positive changes experienced by female thyroid cancer survivors following their struggle with cancer (Cann et al., 2010). The PTGI-SF comprised 10 items categorized into five subscales: Relating to others, new possibilities, personal strength, spiritual change, and appreciation of life. Responses are rated on a 6-point Likert scale, ranging from 0 to 5. The total score of PTGI-SF ranges from 0 to 50, with higher scores indicating greater PTG. In the Chinese population, the PTGI-SF showed acceptable psychometric properties, as indicated by a Cronbach's α value of 0.86 for the whole scale and 0.58–0.79 for the subscales (Liu et al., 2018).

### 2.4 Data collection

The online questionnaire method was adopted in this study to collect data. Throughout the study duration, the QR code for the digital questionnaire was sent to eligible postoperative female thyroid cancer patients and outpatient review participants via an electronic follow-up platform. The online questionnaire's homepage features study's purpose, procedures, risks, benefits, and confidentiality measures. The questionnaire submission is programmed to occur only after all questions have been answered.

### 2.5 Statistical analysis

Data management and analyses were carried out using SPSS 25.0 in this study. To provide an overview of participant characteristics, descriptive statistics was utilized, with categorical variables presented in frequencies, and continuous variables reported as means and standard deviation (SD). The difference of PTG levels among groups was tested by *t*-test or one-way ANOVA. Then, Pearson correlation analysis was performed to examine the relationships between perceived social support, psychological resilience, coping strategies, and PTG. To investigate the mediating roles of psychological resilience and coping strategies, AMOS 26.0 was employed for mediation model analysis. Indirect effects were determined through the bias-corrected bootstrapping procedure with 5,000 bootstrap samples. A mediating effect was deemed significant if the 95 % confidence interval (CI) of effect sizes did not span zero. The model's goodness of fit was assessed based on the following criteria: chi-square ratio to degrees of freedom (χ^2^/df) < 5.0, root mean square error of approximation (RMSEA) < 0.08, goodness-of-fit index (GFI) > 0.90, incremental fit index (IFI) > 0.90, Tucker-Lewis index (TLI) > 0.90, and comparative fit index (CFI) > 0.90 (Wu, 2010). A two-tailed *p*-value < 0.05 was regarded as statistically significant.

### 2.6 Ethical considerations

The study was approved by the Ethics Committee of National Cancer Center/Cancer Hospital, Chinese Academy of Medical Sciences and Peking Union Medical College (Approved Number: 23/543-4286). Informed consent was obtained electronically from all participants. Prior to data collection, participants were presented with a cover page detailing the study's purpose, procedures, risks, benefits, and confidentiality measures. They were explicitly informed that participation was voluntary and that they could withdraw at any time. Electronic consent was secured by requiring participants to select an “I agree” button after reading the information, affirming their voluntary and informed decision to participate. Only upon selecting this option did they proceed to the questionnaire.

## 3 Results

### 3.1 Characteristics of the participants

A total of 635 female thyroid cancer survivors, with a mean age of 41.67 years (SD 10.83) (Table 1), were included in this study. Among them, 395 (62.2 %) were aged 18–44 years. Approximately half of the participants (*n* = 322, 50.7 %) were diagnosed in the past 6 months. Almost all participants (*n* = 626, 98.6 %) were diagnosed with thyroid papillary cancer. Five hundred and seventy-five (90.6 %) participants underwent their surgery once, of which 93.4 % (*n* = 537) were classified as stage I according to the 8th edition of the American Joint Committee on Cancer (AJCC) staging criteria for thyroid cancer.

### 3.2 Descriptive statistics and correlations among variables

The mean total score for perceived social support was 17.51 ± 5.29, with 261 (41.1 %) participants reporting low perceived social support. Psychological resilience had a mean total score of 19.67 ± 2.87 (Supplementary Table 2), with 57 (9.0 %) participants reporting low and 40 (6.3 %) reporting high psychological resilience. Regarding coping strategies, the mean total scores for problem-focused, emotion-focused, and avoidant coping strategies were 15.38 ± 3.94, 23.12 ± 5.66, and 20.95 ± 5.22, respectively (Supplementary Table 3). The mean total score for PTG was 28.66 ± 12.80, with the subscales scores ranging from 5.09 (spiritual change) to 6.15 (personal strength) (Supplementary Table 1).

The correlation analysis revealed that perceived social support was positively correlated with psychological resilience (*r* = 0.290, *p* < 0.001), problem-focused coping strategies (*r* = 0.570, *p* < 0.001), emotion-focused coping strategies (*r* = 0.549, *p* < 0.001), and PTG (*r* = 0.441, *p* < 0.001) (Table 2). Psychological resilience was also positively associated with problem-focused (*r* = 0.208, *p* < 0.001) and emotion-focused coping strategies (*r* = 0.244, *p* < 0.001) and PTG (*r* = 0.130, *p* < 0.01), while negatively associated with dysfunction coping strategies (*r* = −0.099, *p* < 0.05). Additionally, all three coping strategies were positively correlated with PTG (all *p* < 0.01).

**Table 2:** Descriptive statistics and Pearson correlation analysis of post-traumatic growth, social support, psychological resilience, and coping strategies.

**Items**	**Mean ±SD**	**1**	**2**	**3**	**4**	**5**	**6**	**7**	**8**	**9**	**10**	**11**
1. PTG-total	28.66 ± 12.80	1										
2. PTG-relating to others	5.72 ± 2.86	0.913^***^	1									
3. PTG-new possibilities	5.95 ± 2.91	0.942^***^	0.829^***^	1								
4. PTG-personal strength	6.15 ± 3.03	0.911^***^	0.847^***^	0.826^***^	1							
5. PTG-spiritual change	5.09 ± 2.76	0.868^***^	0.724^***^	0.778^***^	0.722^***^	1						
6. PTG-appreciation of life	5.74 ± 2.72	0.843^***^	0.677^***^	0.778^***^	0.664^***^	0.669^***^	1					
7. Perceived social support	17.51 ± 5.29	0.441^***^	0.443^***^	0.415^***^	0.408^***^	0.314^***^	0.389^***^	1				
8. Psychological resilience	19.67 ± 2.87	0.130^**^	0.126^**^	0.138^**^	0.170^***^	0.056	0.087^*^	0.290^***^	1			
9. Problem-focused coping	15.38 ± 3.94	0.483^***^	0.449^***^	0.461^***^	0.421^***^	0.403^***^	0.431^***^	0.570^***^	0.208^***^	1		
10. Emotion-focused coping	23.12 ± 5.66	0.444^***^	0.389^***^	0.407^***^	0.374^***^	0.432^***^	0.388^***^	0.549^***^	0.244^***^	0.801^***^	1	
11. Avoidant coping	20.95 ± 5.22	0.131^**^	0.121^**^	0.093^*^	0.054	0.207^***^	0.118^**^	0.057	−0.099^*^	0.340^***^	0.419^***^	1

PTG, post-traumatic growth; SD, standard difference.

^*^p < 0.05, ^**^p < 0.01, ^***^p < 0.001.

### 3.3 Direct effects among variables

**Figure 1:**
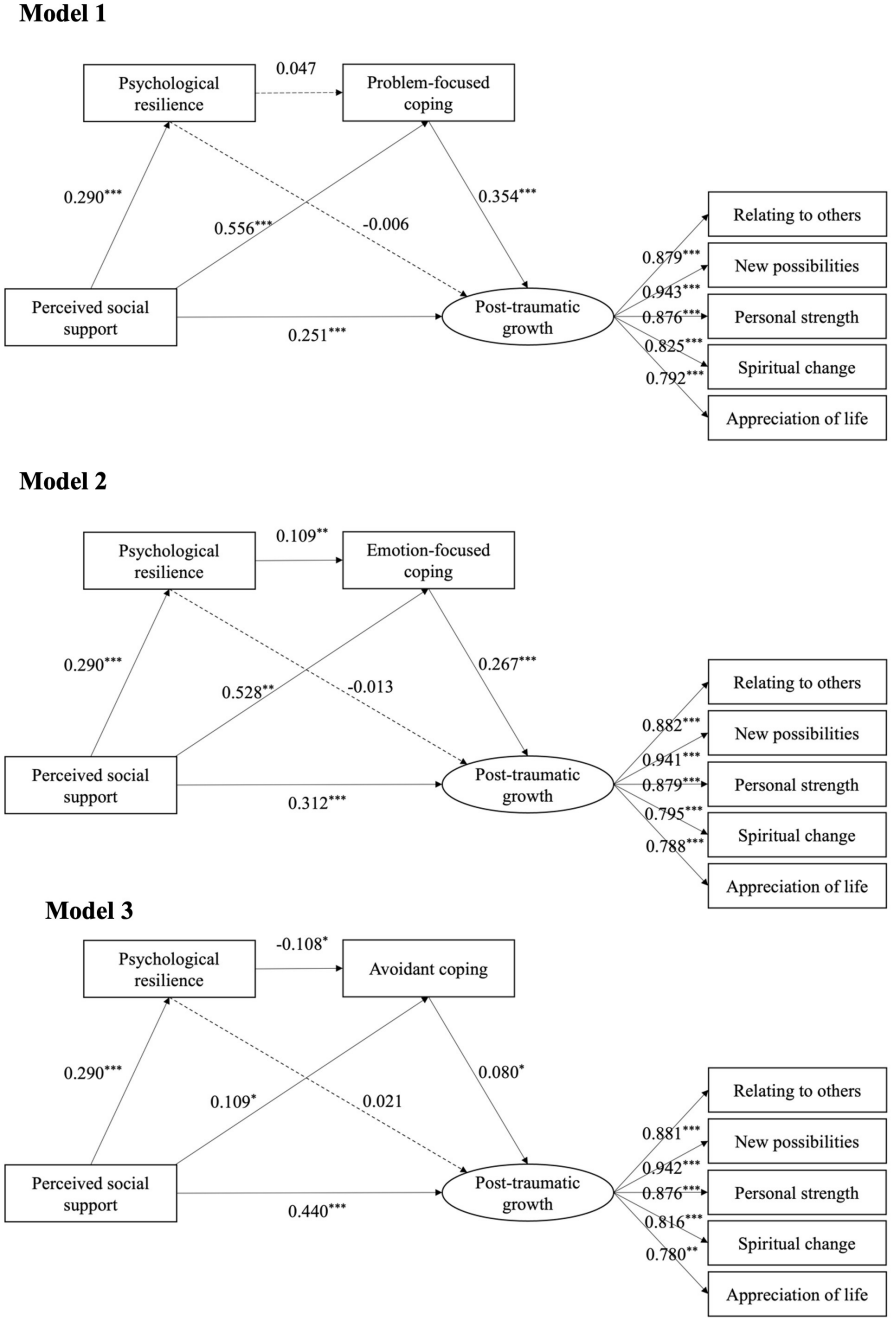
The chain mediating model. Model 1: The chain mediating model of psychological resilience and problem-focused coping on perceived social support and post-traumatic growth. Model 2: The chain mediating model of psychological resilience and emotion-focused coping on perceived social support and post-traumatic growth. Model 3: The chain mediating model of psychological resilience and avoidant coping on perceived social support and post-traumatic growth. *p < 0.05, **p < 0.01, ***p < 0.001.

As depicted in Figure 1, perceived social support emerged as a significant predictor of psychological resilience (*p* < 0.001), problem-focused coping strategies (β = 0.556, *p* < 0.001), emotion-focused coping strategies (β = 0.528, *p* < 0.01), avoidant coping strategies (β = 0.109, *p* < 0.05), and PTG (*p* < 0.001). Moreover, psychological resilience was found to exert a significant positive influence on emotion-focused coping strategies (β = 0.109, *p* < 0.01) while negative influence on avoidant coping strategies (β = −0.108, *p* < 0.05), which in turn positively predicted PTG (*p* < 0.05). However, the direct effect of psychological resilience on PTG was not statistically significant (*p* > 0.05).

### 3.4 Total effect and mediating effects of psychological resilience and coping strategies between perceived social support and PTG

Figure 1 presents the mediating models examing the relationship between perceived social support and PTG, all of which showed acceptable model fit (Table 3). The bootstrap analysis of mediating effects indicated that perceived social support significantly predicted PTG, with total effects of 0.451 (95 %CI 0.368–0.526) in Model 1, 0.457 (95 % CI 0.375–0.532) in Model 2, and 0.452 (95 % CI 0.370–0.528) in Model 3 (Table 4), thus supporting hypothesis 1. Specifically, the direct effects of perceived social support on PTG were 0.251 (95 % CI 0.142–0.353) in Model 1, 0.312 (95 % CI 0.207–0.412) in Model 2, and 0.440 (95 % CI 0.354–0.523) in Model 3. Additionally, the results revealed that psychological resilience and coping strategies partially mediate the effect of perceived social support on PTG, with the total indirect effects being 0.200 (95 % CI 0.136–0.271) in Model 1, 0.145 (95 % CI 0.087–0.212) in Model 2, and 0.012 (95 % CI −0.015–0.040) in Model 3. This included a single mediating pathway from perceived social support to problem-focused/emotion-focused/avoidant coping strategies to PTG (indirect effect size of 0.197 in Model 1, 0.141 in Model 2, and 0.009 in Model 3, hypothesis 3), and a chain mediating pathway from perceived social support to psychological resilience to emotion-focused/avoidant coping strategies to PTG (indirect effect size of 0.008 in Model 2 and −0.003 in Model 3, hypothesis 4). The bootstrap 95 % CI did not include zero for the above two indirect effects, confirming that the above two mediating effects were significant (*p* < 0.05). However, the mediation pathway from perceived social support to psychological resilience to PTG was not significant in any of the models (*p* > 0.05), leading to the rejection of hypothesis 2.

**Table 3:** Model fit summary.

**Models**	**χ^2^/df**	**RMSEA**	**GFI**	**IFI**	**TLI**	**CFI**
Model 1	3.162	0.058	0.983	0.991	0.982	0.991
Model 2	2.970	0.056	0.985	0.993	0.984	0.992
Model 3	3.574	0.064	0.981	0.989	0.977	0.989

**Table 4:** Effect sizes and Bootstrap analysis results of mediating effect.

**Models**	**Mediating effect**	**Pathway**	**Standard effect value**	**Boot standard error**	**BootLICI**	**BootULCI**	***p*-value**
Model 1	Total effect		0.451	0.040	0.368	0.526	< 0.001
	Direct effect	Perceived social support → PTG	0.251	0.053	0.142	0.353	< 0.001
	Indirect effect	Perceived social support → Problem-focused coping → PTG	0.197	0.032	0.138	0.262	< 0.001
		Perceived social support → Psychological resilience → PTG	−0.002	0.013	−0.028	0.021	0.915
		Perceived social support → Psychological resilience → Problem-focused coping → PTG	0.005	0.004	−0.003	0.015	0.191
	Total indirect effect		0.200	0.034	0.136	0.271	< 0.001
Model 2	Total effect		0.457	0.040	0.375	0.532	< 0.001
	Direct effect	Perceived social support → PTG	0.312	0.052	0.207	0.412	< 0.001
	Indirect effect	Perceived social support → Emotion-focused coping → PTG	0.141	0.028	0.089	0.201	< 0.001
		Perceived social support → Psychological resilience → PTG	−0.004	0.013	−0.031	0.019	0.760
		Perceived social support → Psychological resilience → Emotion-focused coping → PTG	0.008	0.003	0.003	0.017	0.002
	Total indirect effect		0.145	0.031	0.087	0.212	< 0.001
Model 3	Total effect		0.452	0.040	0.370	0.528	< 0.001
	Direct effect	Perceived social support → PTG	0.440	0.042	0.354	0.523	< 0.001
	Indirect effect	Perceived social support → Avoidant coping → PTG	0.009	0.006	0.000	0.025	0.039
		Perceived social support → Psychological resilience → PTG	0.006	0.013	−0.020	0.031	0.595
		Perceived social support → Psychological resilience → Avoidant coping → PTG	−0.003	0.002	−0.007	0.000	0.031
	Total indirect effect		0.012	0.014	−0.015	0.040	0.349

PTG, post-traumatic growth; Boot LLCI and Boot ULCI, the lower and upper limits of the 95 % confidence interval of the effects estimated by the percentile bootstrap method with deviation correction.

## 4 Discussion

### 4.1 Main findings

PTG has been extensively studied in cancer patients and survivors, demonstrating its contribution to improved quality of life. However, the research into PTG in female thyroid cancer survivors has been relatively limited. This study addressed this gap by examining the status and the underlying mechanisms of PTG in female thyroid cancer survivors. The findings revealed that female thyroid cancer survivors had an average PTG score of 28.66 ± 12.80, which was directly influenced by perceived social support and indirectly influenced through both single mediating effects involving problem-focused/emotion focused/avoidant coping strategies and a chain mediating effect involving psychological resilience and emotion focused/avoidant coping strategies.

The findings of the present study underscore the significant role of perceived social support in predicting PTG among female thyroid cancer survivors. This aligns with prior research conducted on patients and survivors of other types of cancers, such as breast cancer and colorectal cancer (Dong et al., 2017; Ofei et al., 2023). Perceived social support impacts PTG in multiple ways. Firstly, external social support creates a supportive environment that encourages open communication and cognitive reappraisal and provides fresh perspectives (Mah et al., 2024), ultimately promoting individuals into deliberate rumination and fostering PTG (Tedeschi and Calhoun, 2004). Additionally, perceived social support can indirectly influence PTG by serving as a buffer against distress (Cohen and Wills, 1985). Specifically, when cancer patients encounter stress or challenges, perceived support from the community can bolster them, thereby attenuating the adverse impact of such stressors on their mental wellbeing (Applebaum et al., 2014; Haugland et al., 2016). Therefore, ensuring adequate social support for female thyroid cancer survivors, particularly during their diagnosis and treatment journey, plays a pivotal role in fostering their PTG.

This study revealed that coping strategies played a significant mediating role in the relationship between perceived social support and PTG in female thyroid cancer survivors. This result was partially supported by a recent systematic review which identified social support as a predictor of coping strategies among cancer patients (Bottaro et al., 2023). Moreover, coping strategies have been highlighted as pivotal in the original growth model proposed by Tedeschi and Calhoun for the development of PTG (Tedeschi and Calhoun, 2004). Collectively, it could be inferred that perceived social support may influence PTG through coping strategies in female thyroid cancer survivors. This can be reasoned as follows: perceived social support from family, friends, healthcare professionals, and other sources may encourage patients to adopt positive and effective coping strategies to navigate the challenges and stresses inherent in cancer treatment (Calderon et al., 2021; Mishra and Saranath, 2019), such as seeking support, acquiring information, and regulating emotions. By utilizing positive coping strategies, patients can more effectively navigate the challenges and impacts of their illness, fostering personal growth and development while facilitating the progression of PTG. Notably, a longitudinal study has shown the enduring effects of positive coping strategies on PTG in breast cancer survivors 6 months and 2 years post-surgery (Hamama-Raz et al., 2019). Consequently, coping strategy training emerges as a promising intervention to enhance PTG among female thyroid cancer survivors.

A novel finding of the present study was the identification of a chain mediating role played by psychological resilience and emotion-focused/avoidant coping strategies in the relationship between perceived social support and PTG among female thyroid cancer survivors. However, psychological resilience did not exhibit a direct significant mediating effect between social support and PTG. This suggests that while psychological resilience serves as an essential internal resource when confronting trauma, it facilitates PTG primarily through indirect pathways, specifically by empowering individuals to adopt more adaptive and proactive coping strategies such as seeking social support and engaging in positive reappraisal. This may be explained by the treatment cycle of thyroid cancer. Generally, thyroid cancer treatment involves a relatively short clinical course but distinct and prolonged psychological stressors, patients may become particularly dependent on their selected coping mechanisms during the early post-operative adjustment phase, while psychological resilience provides the internal foundation and support for the adaptive behaviors. Therefore, clinical interventions should aim not only to strengthen psychological resilience, but also to provide specific coping strategies tailored to address issues such as body image and fertility-related distress.

Additionally, it is important to acknowledge that existing research has reported mixed results regarding the direction of the relationship between psychological resilience and coping strategies. For example, in alignment with our findings, a previous study showed that resilience level directly and indirectly predicted PTG or post-traumatic symptoms among cancer patients, with different coping strategies as mediators (Gori et al., 2021). Conversely, a recent study among Korean female cancer patients suggested that problem-focused coping strategies might bolster resilience, consequently fostering PTG (Choi et al., 2023). Hence, further research is warranted to explore the causal relationship between psychological resilience and coping strategies. Nonetheless, the chain mediating model uncovered in our study contributes to the development of interventions aimed at enhancing PTG in female thyroid cancer survivors, and potentially benefiting other cancer patients and survivors as well.

### 4.2 Clinical implications

Given the significant impact of perceived social support on PTG among female thyroid cancer survivors, healthcare professionals should recognize its crucial role and help individuals access tailored resources to address their specific needs. These resources may include support from partners, empathy, family caregiving, and information provision (Korotkin et al., 2019), all of which contribute to improved mental wellbeing and overall quality of life. In particular, regarding information provision, healthcare providers should not only supply disease-related knowledge, treatment options, and side-effect management guidance, but also offer early fertility counseling to reduce reproductive anxieties (Benedict et al., 2020; Wang et al., 2021), thereby promoting PTG. In addition, incorporating psychological resilience training, such as cognitive-behavioral therapy and mindfulness practices aiming to enhance acceptance of bodily changes resulting from treatment (e.g., surgical scars or hormone therapy), could also aid patients in effectively managing stress and trauma. Similarly, implementing coping strategy training programs offers another viable pathway to foster PTG. For example, expressive arts (e.g., painting or body mapping) can facilitate the processing of negative emotions, while practical skills workshops (e.g., clothing coordination techniques) can assist in scar camouflage, both contributing to the development of PTG.

### 4.3 Strengths and limitations

To the best of our knowledge, this study represents the first exploration of the chain mediating role of psychological resilience and coping strategies between perceived social support and PTG in female thyroid cancer survivors. The encouraging findings from this study shed light on the underlying mechanisms by which perceived social support influences PTG in female thyroid cancer survivors. Furthermore, this study particularly focuses on female thyroid cancer survivors to enhance the accuracy of the research conclusions, considering the differences in coping mechanisms and psychological adjustment between males and females (Hasan et al., 2022). The findings from this study may also have broader applicability to other female cancer patient cohorts.

Nevertheless, it should be noted that this study also has several limitations. First, the use of an online survey to collect data may introduce selective bias in the sample and result in lower response rates, as only those with internet access could participate. Second, the study sample comprised solely female thyroid cancer survivors, which may limit the generalizability of the findings to male thyroid cancer survivors, who have been reported to experience lower levels of PTG compared to females (Powroznik et al., 2018). Third, the chain mediation model was developed using cross-sectional data, the nature of which limited our ability to establish causality between perceived social support, psychological resilience, coping strategies, and PTG. Future studies are recommended to use longitudinal designs to ascertain the causality between variables to validate our results and provide a comprehensive understanding and insights for enhancing PTG in female thyroid cancer survivors.

## 5 Conclusions

In conclusion, this study illuminates the potential pathways through which perceived social support influences PTG in female thyroid cancer survivors. The findings suggest that perceived social support not only directly impacts PTG but also influences psychological resilience and coping strategies in this population. Furthermore, a noteworthy contribution of this study is the identification of psychological resilience and emotion-focused/avoidant coping strategies as chain mediators in the relationship between perceived social support and PTG among female thyroid cancer survivors. These findings offer valuable insights for interventions aimed at fostering PTG in female cancer patients and survivors. Strategies may include the provision of social support, enhancement of psychological resilience, and encouragement of the adoption of positive coping strategies.

## Data Availability

The raw data supporting the conclusions of this article will be made available by the authors, without undue reservation.
