# Supplementary material for: Association between perceived social support and post-traumatic growth among female thyroid cancer survivors: the chain mediating role of psychological resilience and coping strategies
Source: Front Psychol. 2025 Oct 6;16:1614974. doi: 10.3389/fpsyg.2025.1614974 (PMC12535981; doi:10.3389/fpsyg.2025.1614974)
Supplement: Supplementary file 1 [file Table_1.docx]

**Table A.1 Descriptive statistics of post-traumatic growth.**

| Items | Range | Mean ± SD |
| --- | --- | --- |
| Relating to others | 0-10 | 5.72 ± 2.856 |
| New possibilities | 0-10 | 5.95 ± 2.909 |
| Personal strength | 0-10 | 6.15 ± 3.033 |
| Spiritual change | 0-10 | 5.09 ± 2.759 |
| Appreciation of life | 0-10 | 5.74 ± 2.724 |
| Total | 0-50 | 28.66 ± 12.798 |

**Note:** SD, standard difference.

**Table A.2 Descriptive statistics of psychological resilience.**

| Items | Range | Mean ± SD |
| --- | --- | --- |
| I tend to bounce back quickly after hard times. | 1-5 | 3.93 ± 0.870 |
| I have a hard time making it through stressful events. | 1-5 | 2.77 ± 1.104 |
| It does not take me long to recover from a stressful event. | 1-5 | 3.72 ± 0.929 |
| It is hard for me to snap back when something bad happens. | 1-5 | 2.85 ± 1.088 |
| I usually come through difficult times with little trouble. | 1-5 | 3.54 ± 0.917 |
| I tend to take a long time to get over set-backs in my life. | 1-5 | 2.86 ± 1.041 |
| Total score | 6-30 | 19.67 ± 2.866 |
| Average item score | 1-5 | 3.28 ± 0.478 |

**Note:** SD, standard difference.

**Table A.3 Descriptive statistics of coping strategies.**

| Items | Range | Mean ± SD |
| --- | --- | --- |
| Emotion-focused strategies | 10-40 | 23.12 ± 5.662 |
| Use of emotional support | 2-8 | 4.47 ± 1.610 |
| Positive reframing | 2-8 | 5.30 ± 1.585 |
| Acceptance | 2-8 | 5.92 ± 1.682 |
| Religion | 2-8 | 3.53 ± 1.481 |
| Humor | 2-8 | 3.90 ± 1.617 |
| Problem-focused strategies | 6-24 | 15.38 ± 3.937 |
| Active coping | 2-8 | 5.65 ± 1.686 |
| Planning | 2-8 | 5.13 ± 1.582 |
| Use of instrumental support | 2-8 | 4.60 ± 1.498 |
| Dysfunctional coping strategies | 12-48 | 20.95 ± 5.222 |
| Venting | 2-8 | 4.22 ± 1.254 |
| Denial | 2-8 | 3.13 ± 1.406 |
| Substance use | 2-8 | 2.44 ± 1.047 |
| Behavioral disengagement | 2-8 | 3.17 ± 1.388 |
| Self-distraction | 2-8 | 4.44 ± 1.232 |
| Self-blame | 2-8 | 3.55 ± 1.250 |

**Note:** SD, standard difference.
